# Copper‐Free Synthesis of Cationic Glycidyl Triazolyl Polymers

**DOI:** 10.1002/marc.202400416

**Published:** 2024-07-02

**Authors:** Taichi Ikeda

**Affiliations:** ^1^ 1‐1 Namiki Tsukuba Ibaraki 305‐0044 Japan

**Keywords:** glycidyl triazolyl polymers, ion conductive materials, microwave reaction, poly(ionic liquid)s, polyelectrolytes

## Abstract

Copper‐free synthesis of cationic glycidyl triazolyl polymers (GTPs) is achieved through a thermal azide‐alkyne cycloaddition reaction between glycidyl azide polymer and propiolic acid, followed by decarboxylation and quaternization of the triazole unit. For synthesizing nonfunctionalized GTP (GTP‐H), a microwave‐assisted method enhances the decarboxylation reaction of carboxy‐functionalized GTP (GTP‐COOH). Three variants of cationic GTPs with different *N*‐substituents [*N*‐ethyl, *N*‐butyl, and *N*‐tri(ethylene glycol) monomethyl ether (EG3)] are synthesized. The molecular weight of GTP‐H is determined via size exclusion chromatography. Thermal properties of all GTPs are characterized using differential scanning calorimetry and thermogravimetric analysis. The ionic conductivities of these cationic GTPs are assessed by impedance measurements. The conducting ion concentration and mobility are calculated based on the electrode polarization model. Among three cationic GTPs, the GTP with the *N*‐EG3 substituent exhibits the highest ionic conductivity, reaching 6.8 × 10^−6^ S cm^−1^ at 25 °C under dry conditions. When compared to previously reported reference polymers, the reduction of steric crowding around the triazolium unit is considered to be a key factor in enhancing ionic conductivity.

## Graphical abstract

Cationic glycidyl triazolyl polymers are synthesized via thermal azide‐alkyne cycloaddition, followed by decarboxylation and quaternization of the triazole unit. A microwave‐assisted reaction enhances the efficiency of the decarboxylation step. The polymers featuring ethylene glycol side chains exhibit superior ionic conductivity, achieving 6.8 × 10^−6^ S cm^−1^ at 25 °C under dry conditions.

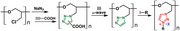

## 1 Introduction

Polymer electrolytes based on poly(ionic liquid)s are valuable materials across a range of applications, including batteries,^[^ ^1^, ^2^ ^]^ fuel cells,^[^ ^3^, ^4^ ^]^ supercapacitors,^[^ ^5^, ^6^, ^7^ ^]^ solar cells,^[^ ^8^, ^9^ ^]^ electrochromic devices,^[^ ^10^, ^11^ ^]^ actuators,^[^ ^12^, ^13^ ^]^ CO_2_ absorption,^[^ ^14^, ^15^ ^]^ and separation membranes,^[^ ^16^, ^17^ ^]^ owing to their excellent processability and high design flexibility. While most research groups have focused on developing acrylate polymer‐based poly(ionic liquid)s through the polymerization of ionic liquid monomers,^[^ ^18^, ^19^, ^20^, ^21^ ^]^ our group has explored poly(ionic liquid)s based on glycidyl triazolyl polymers (GTPs).^[^ ^22^, ^23^, ^24^, ^25^, ^26^, ^27^ ^]^ These GTPs are synthesized via a Cu(I)‐catalyzed azide‐alkyne cycloaddition reaction between glycidyl azide polymer (GAP) and cationic or anionic alkyne derivatives. A significant advantage of our GTP‐based poly(ionic liquid)s is their post‐functionalization capability, allowing for the preparation of a series with consistent degrees of polymerization to elucidate the structure–property relationships.^[^ ^22^, ^27^, ^28^ ^]^ However, the use of the copper catalyst requires a tedious and time‐consuming work‐up procedure for purification. To address this issue, a new synthetic route for GTP‐based poly(ionic liquid)s without a copper catalyst has been developed in this study, which involves: i) thermal azide‐alkyne cycloaddition between GAP and propiolic acid to produce carboxy‐functionalized GTP (GTP‐COOH), ii) synthesis of nonfunctionalized GTP (GTP‐H) through decarboxylation of GTP‐COOH, and iii) synthesis of cationic GTP through quaternization of the triazole group. These first two steps were originally reported by H. L. Cohen in 1981.^[^ ^29^ ^]^ Although Cohen mentioned the synthesis of GTP‐H as an example of the chemical modification of various azide polymers, the details of the synthetic procedure and characterization results were not reported beyond CHN elemental analysis data. There is no further chemical and physical data on GTP‐H because nobody revisited his achievement since 1981. In this study, we have successfully replicated Cohen's methodology and refined the synthetic procedure, particularly finding that microwave‐assisted reaction efficiently promotes the decarboxylation of GTP‐COOH.

**Figure 1:**
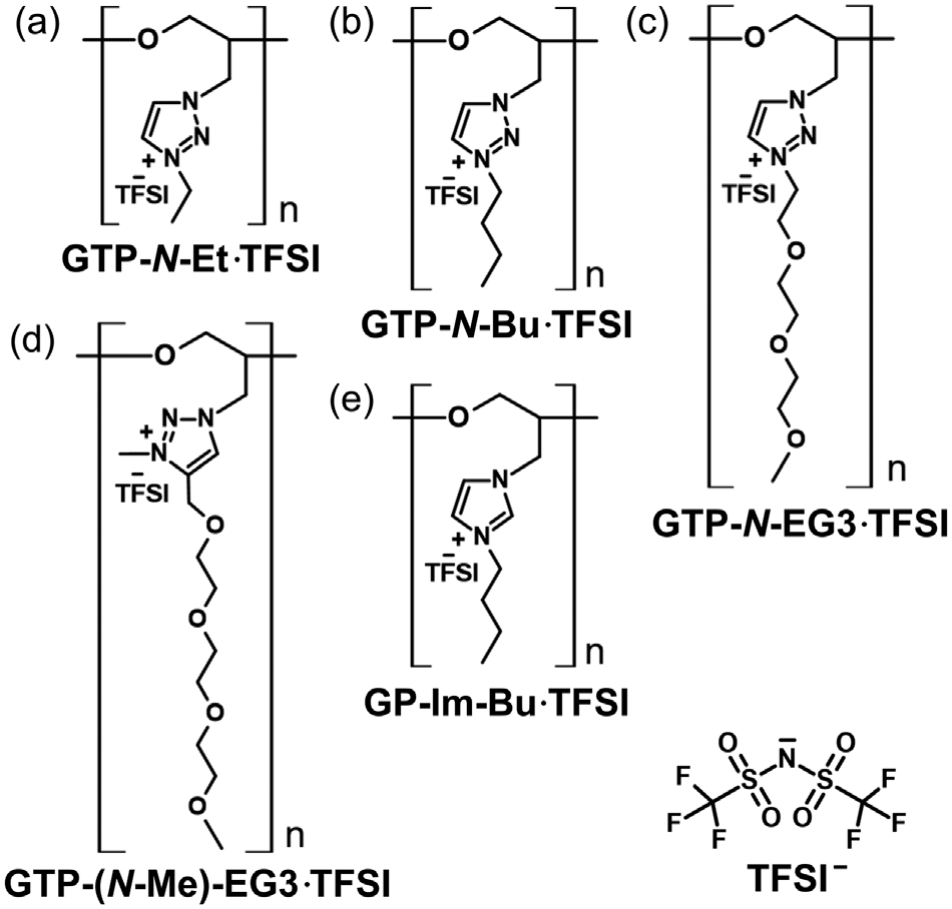
a–c) Chemical structures of cationic GTPs in this study, and d,e) reference polymers in previous studies.

In this study, three types of cationic GTPs with different *N*‐substitutions [‐ethyl, butyl, and tri(ethylene glycol) monomethyl ether] (**Figure** **1**a–c) were synthesized, and their thermal properties and ionic conductivity were characterized. The triazolium‐based poly(ionic liquid)s have been extensively reported.^[^ ^30^, ^31^, ^32^, ^33^, ^34^, ^35^, ^36^ ^]^ Among these, GTP‐(*N*‐Me)‐EG3·TFSI (Figure 1d) served as a reference polymer, synthesized through a copper(I)‐catalyzed azide‐alkyne cycloaddition.^[^ ^36^ ^]^ In addition, imidazolium‐based poly(ionic liquid) with a glycidyl main chain (GP‐Im‐Bu·TFSI) was also selected as a reference polymer for comparison (Figure 1e).^[^ ^37^ ^]^ It was found that GTP‐*N*‐Bu·TFSI exhibited ionic conductivity comparable to GP‐Im‐Bu·TFSI, while GTP‐*N*‐EG3·TFSI showed higher ionic conductivity than GTP‐(*N*‐Me)‐EG3·TFSI. The influence of the substituents on the triazole unit on ionic conductivity is discussed in this study.

## 2 Results and Discussion

### 2.1 Synthesis of Cationic GTPs

**Scheme 1:**
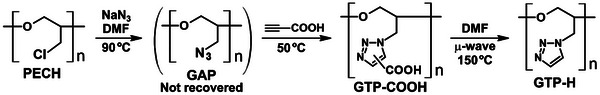
Synthetic route of non‐functionalized GTP.

The synthetic route of GTP‐H is illustrated in **Scheme** **1**. Polyepichlorohydrin (PECH) was reacted with sodium azide (NaN_3_) in DMF at 90 °C for 24 h. This reaction condition led to 100% conversion from PECH to GAP.^[^ ^22^, ^23^, ^24^, ^25^, ^26^, ^27^ ^]^ In our previous studies, GAP was precipitated by gradually adding the reaction solution to water.^[^ ^22^, ^23^, ^24^, ^25^, ^26^, ^27^ ^]^ However, for safety reasons, it is preferable to proceed to the next reaction without isolating GAP in solid form, due to its high‐energy content and associated explosion risk.^[^ ^38^ ^]^ H. L. Cohen demonstrated that subsequent reactions could be conducted directly after removing the salts (NaCl and unreacted NaN_3_) from the solution by filtration. It was confirmed that this approach was effective, yielding a white product after reacting with propiolic acid in DMF at 50 °C for 3 d. The quantitative conversion from GAP to GTP‐COOH was confirmed by the disappearance of the azide peak (*ν* = 2100 cm^−1^) in the IR spectrum. While higher reaction temperature reduced the reaction time, it also caused significant discoloration of the solution.

**Figure 2:**
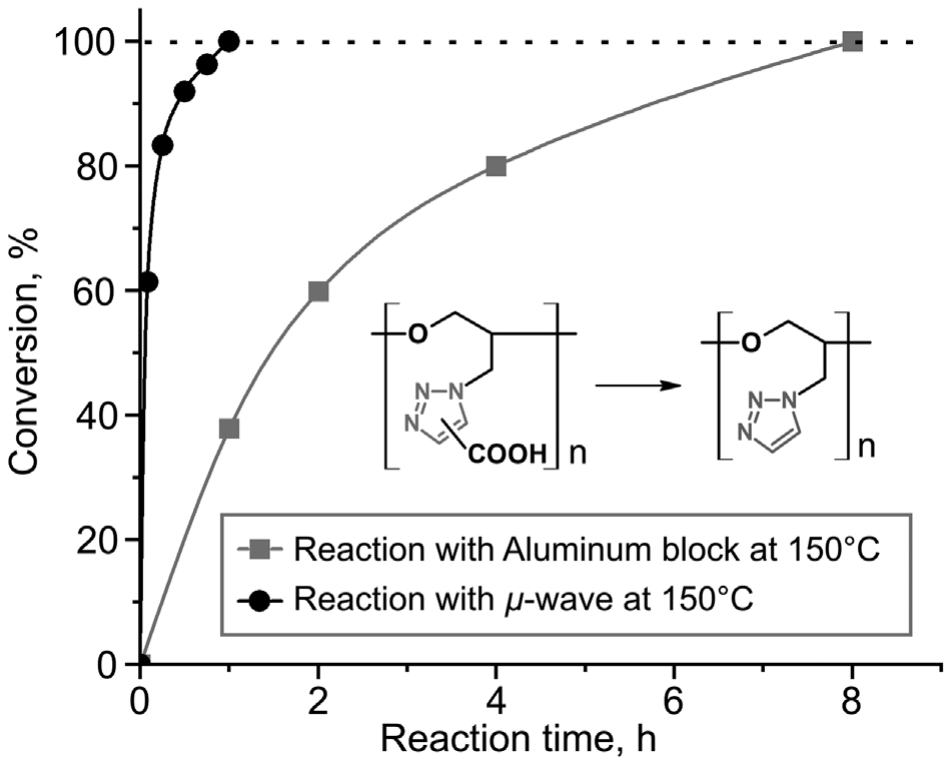
Conversion versus time plot for the decarboxylation reaction of GTP‐COOH. The reaction was conducted in a DMF solution, with the reaction time starting upon reaching 150 °C.

H. L. Cohen performed the decarboxylation of GTP‐COOH at 190 °C in *N*‐methylpyrrolidone (NMP) for 4 h.^[^ ^29^ ^]^ We found that decarboxylation could be conducted at a lower temperature (150 °C) using *N,N*‐dimethylformamide (DMF), which is more cost effective and easier to evaporate than NMP, making this a preferable reaction condition. **Figure** **2** displays the conversion versus time curves for the decarboxylation reaction, determined from the integrals of the triazole peak in the ^1^H NMR spectrum (Figure S10, Supporting Information). Using an aluminum reaction/heating block at 150 °C, the reaction took eight hours to complete. A microwave‐assisted reaction dramatically shortened this time to one hour (Figure 2). The acceleration of the decarboxylation reaction by the microwave irradiation has been reported by some groups.^[^ ^39^, ^40^, ^41^ ^]^ It was hypothesized that the microwave effects would arise from large polarity change between the ground state and the transition state in the decarboxylation reaction.^[^ ^41^, ^42^ ^]^

**Scheme 2:**
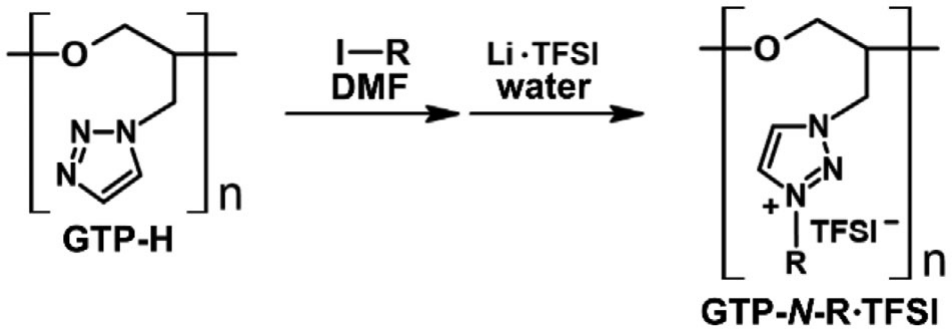
Synthesis of cationic GTPs.

The quaternization of the triazole group was carried out by reacting with iodide compounds (**Scheme** **2**). The reaction conditions are summarized in **Table** **1**. Following the counterion metathesis with lithium bis(trifluoromethanesulfonyl)imide (Li·TFSI), the cationic GTPs were obtained. For the *N*‐ethyl and *N*‐butyl variants, transparent pale‐yellow rubbers were produced. The synthesis of GTP‐*N*‐EG3·TFSI required higher temperatures and longer reaction times, resulting in a transparent orange rubber. Although the conversion of triazole to triazolium groups was complete (100%), the yield was ≈70% due to losses during the precipitation purification process (Table 1).

**Table 1:** Reaction conditions and yields of cationic GTPs.

GTP	R	*T* ^a)^ [°C]	*t* ^b)^ [h]	Yield [%]
GTP‐*N*‐Et·TFSI	C_2_H_5_	70	24	75
GTP‐*N*‐Bu·TFSI	C_4_H_9_	80	24	73
GTP‐*N*‐EG3·TFSI	(C_2_H_4_O)_3_CH_3_	100	48	68

^a)^ Reaction temperature;

^b)^ Reaction time.

**Figure 3:**
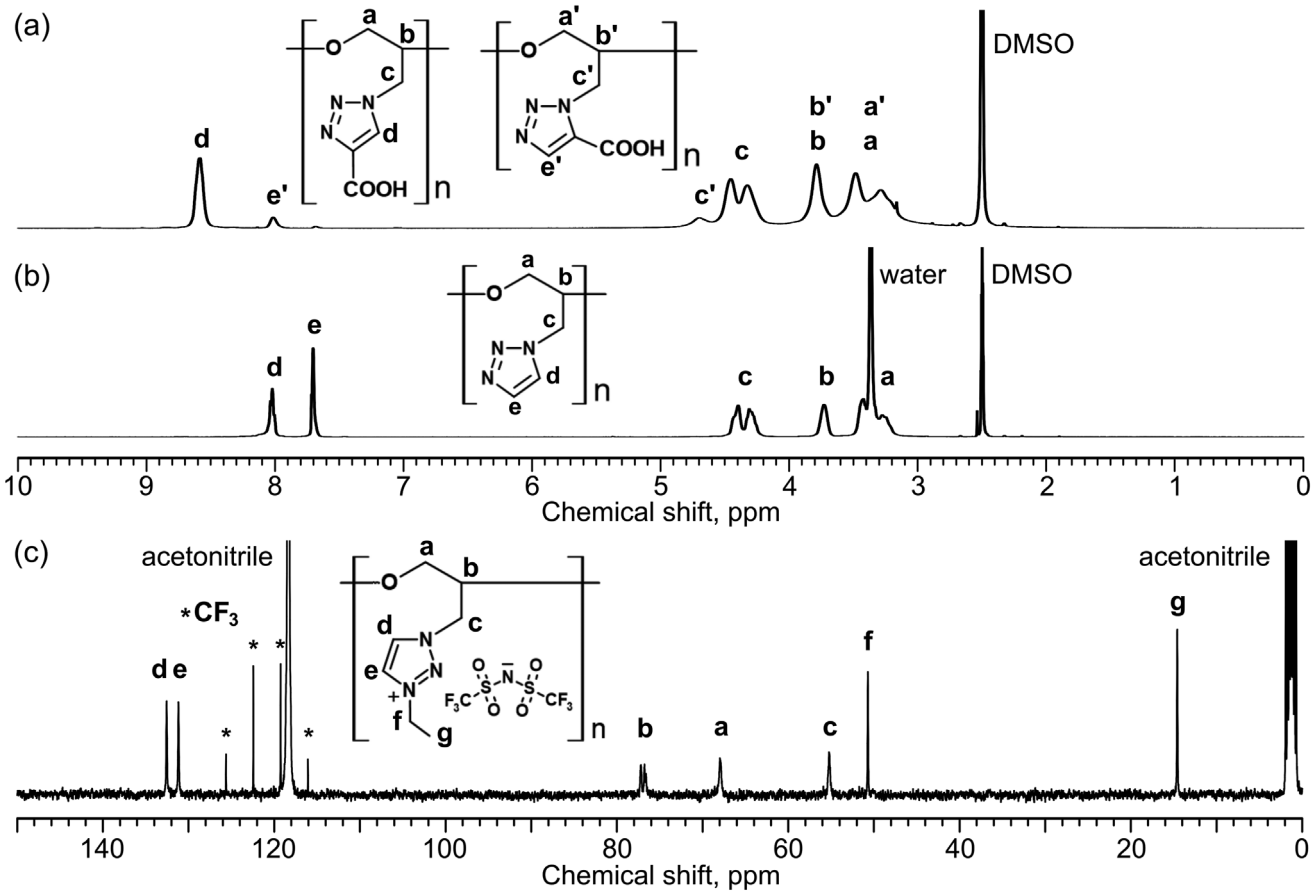
^1^H NMR spectra of a) GTP‐COOH and b) GTP‐H (DMSO‐*d*_6_, 400 MHz); c) ^13^C NMR spectra of GTP‐*N*‐Et·TFSI (CD_3_CN, 100 MHz).

The chemical structures of the products were confirmed using ^1^H, ^13^C, and 2D NMR spectroscopy. **Figure** **3**a presents the ^1^H NMR spectra of GTP‐COOH, where thermal azide‐alkyne cycloaddition results in a mixture of 4‐ and 5‐functionalized triazoles.^[^ ^43^ ^]^ The peaks at 8.58 and 8.00 ppm correspond to the triazole protons of 4‐ and 5‐functionalized products, respectively,^[^ ^43^ ^]^ with a ratio of 9:1 determined from the integrals of these peaks. The integrals of ^1^H NMR peaks are shown in Figures S1–S5 (Supporting Information). The disappearance of water and carboxyl proton peaks is likely due to the intermediate exchange rate via hydrogen bonding on the NMR timescale. Figure 3b illustrates the ^1^H NMR spectra of GTP‐H. Peak assignments were facilitated by ^1^H‐^13^C heteronuclear multiple bond coherence (HMBC) spectroscopy, which indicated a cross‐peak between proton c and carbon d (Figure S7, Supporting Information). The glycidyl protons (a, b, and c) in GTP‐H appear simpler compared to those in GTP‐COOH, due to the absence of structural isomer. Figure 3c displays the ^13^C NMR spectra of GTP‐*N*‐Et·TFSI, confirming the presence of glycidyl (a, b, and c), triazolium (d and e), ethyl group (f and g), and TFSI counter anion carbons. The HMBC spectra showed cross‐peaks between proton c and carbon d, and between proton f and carbon e (Figure S9, Supporting Information). The carbon of the CF_3_ group split into a quartet due to ^13^C–^19^F spin–spin coupling.^[^ ^44^ ^]^ Comparing GTP‐*N*‐EG3·TFSI with the reference polymer GTP‐(*N*‐Me)‐EG3·TFSI (Figure 1d), the triazolium proton peak of GTP‐(*N*‐Me)‐EG3·TFSI (d) is broader than those of GTP‐*N*‐EG3·TFSI (d and e, Figure S11, Supporting Information).^[^ ^36^ ^]^ This broadening is likely due to the different steric environments surrounding the triazolium units in GTP‐*N*‐EG3·TFSI and GTP‐(*N*‐Me)‐EG3·TFSI. The triazolium unit in GTP‐(*N*‐Me)‐EG3·TFSI has two substituents at the 3‐ and 4‐positions, whereas GTP‐*N*‐EG3·TFSI has only one substituent at the 3‐position. The presence of additional substituents in GTP significantly impacts the dynamics of the triazolium unit due to its proximity to the main chain, leading to peak broadening from restricted dynamics with short NMR relaxation time.^[^ ^22^ ^]^

The molecular weight of GTP‐H was determined by size exclusion chromatography (SEC), using polystyrene as a standard. Figure S12 (Supporting Information) presents the SEC chart for GTP‐H. The number‐average and weight‐average molecular weights (*M*_n_ and *M*_w_) were 1.6 × 10^5^ and 2.5 × 10^5^ g mol^−1^, respectively. Attempts to measure the molecular weights of GTP‐COOH and cationic GTPs by SEC were unsuccessful, as no peaks appeared on the SEC chart, likely due to adsorption onto the column resin. **Table** **2** summarizes the *M*_n_ and *M*_w_ values of the GTPs, calculated based on the number‐ and weight‐average degrees of polymerization of GTP‐H.

**Table 2:** Molecular weight and thermal properties of GTPs.

GTP	*M*_n_ ^a)^ [g mol^−1^]	*M*_w_ ^b)^ [g mol^−1^]	*T*_g_ ^c)^ [°C]	*T*_d5_ ^d)^ [°C]
GTP‐COOH^e)^	2.2 × 10^5^ ^f)^	3.4 × 10^5^ ^g)^	110.6	173
GTP‐H^h)^	1.6 × 10^5^	2.5 × 10^5^	47.5	337
GTP‐*N*‐Et·TFSI	5.6 × 10^5^ ^f)^	8.8 × 10^5^ ^g)^	−11.3	350
GTP‐*N*‐Bu·TFSI	5.9 × 10^5^ ^f)^	9.4 × 10^5^ ^g)^	−14.6	340
GTP‐*N*‐EG3·TFSI	7.1 × 10^5^ ^f)^	1.1 × 10^6^ ^g)^	−24.3	325

^a)^ Number‐average molecular weight;

^b)^ Weight‐average molecular weight;

^c)^ Glass transition temperature, onset value of DSC curve;

^d)^ 5 wt% decomposition temperature determined by TGA curve;

^e)^ Mixture of 4‐functionalized and 5‐functionalized triazoles;

^f)^ Calculated value based on number‐average degree of polymerization of GTP‐H;

^g)^ Calculated value based on weight‐average degree of polymerization of GTP‐H;

^h)^ Polydispersity index PDI = 1.6.

### 2.2 Thermal Properties

**Figure 4:**
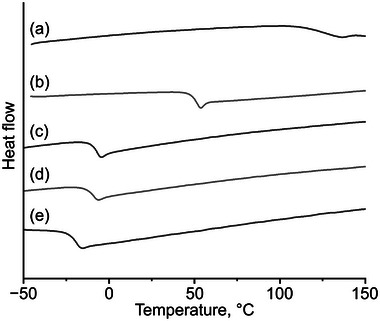
a–e) DSC charts of GTP‐COOH, GTP‐H, GTP‐*N*‐Et·TFSI, GTP‐*N*‐Bu·TFSI, and GTP‐*N*‐EG3·TFSI. Heating rate: 10 °C min^−1^.

Thermal properties were characterized using differential scanning calorimetry (DSC) and thermogravimetric analysis (TGA). **Figure** **4** displays the DSC charts for all GTPs, which exhibited glass transitions without melting or crystallization peaks. The glass transition temperatures (*T*_g_) are detailed in Table 2. The main product of GTP‐COOH in this study is the 4‐functionalized product, although GTP‐COOH is a mixture with the 5‐functionalized variant. Consequently, the *T*_g_ of GTP‐COOH in this study (110.6 °C) is comparable to the previously reported value for GTP‐COOH synthesized with a Cu(I) catalyst (110.8 °C);^[^ ^45^ ^]^ however, the step change at *T*_g_ in Figure 4a was less pronounced than previously reported. The *T*_g_ of GTP‐H was slightly above room temperature at 47.5 °C, and the *T*_g_s of the cationic GTPs were below room temperature, rendering these polymers as adhesive rubber materials. It was confirmed that a longer *N*‐substituent resulted in a lower *T*_g_ value.

**Figure 5:**
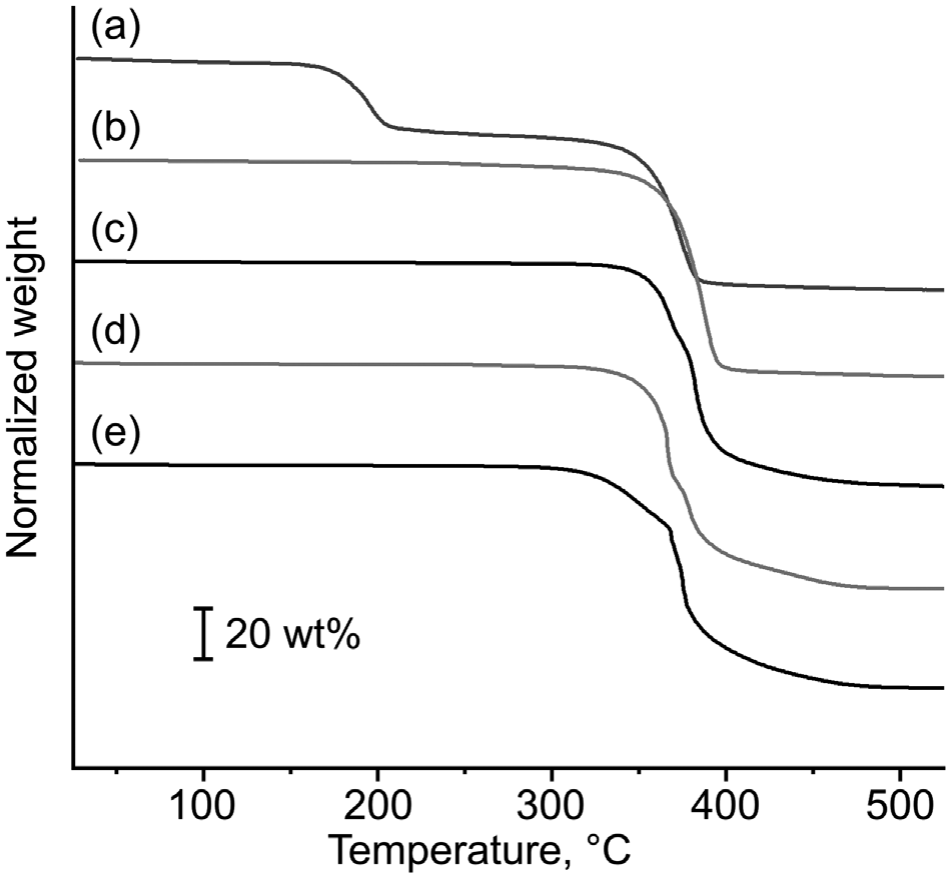
a–e) TGA charts of GTP‐COOH, GTP‐H, GTP‐*N*‐Et·TFSI, GTP‐*N*‐Bu·TFSI, and GTP‐*N*‐EG3·TFSI. Heating rates were set at 5 °C min^−1^ for GTP‐COOH and 10 °C min^−1^ for the others.

**Figure** **5** illustrates the TGA charts. The 5 wt% loss temperatures (*T*_d5_s) are summarized in Table 2. For GTP‐COOH (Figure 5a), a 26 wt% weight loss was observed between 150 and 220 °C, consistent with the expected weight loss from the decarboxylation reaction (monomer unit molecular weights of GTP‐COOH and GTP‐H are 169.14 and 125.13 g mol^−1^, respectively). The second significant weight loss for GTP‐COOH occurred at the same temperature as the thermal decomposition of GTP‐H (Figure 5b), as these are composed of the same polymer base. The *T*_d5_ values of the cationic GTPs were above 300 °C, indicating their thermal stability.

### 2.3 Ionic Conductivity

**Figure 6:**
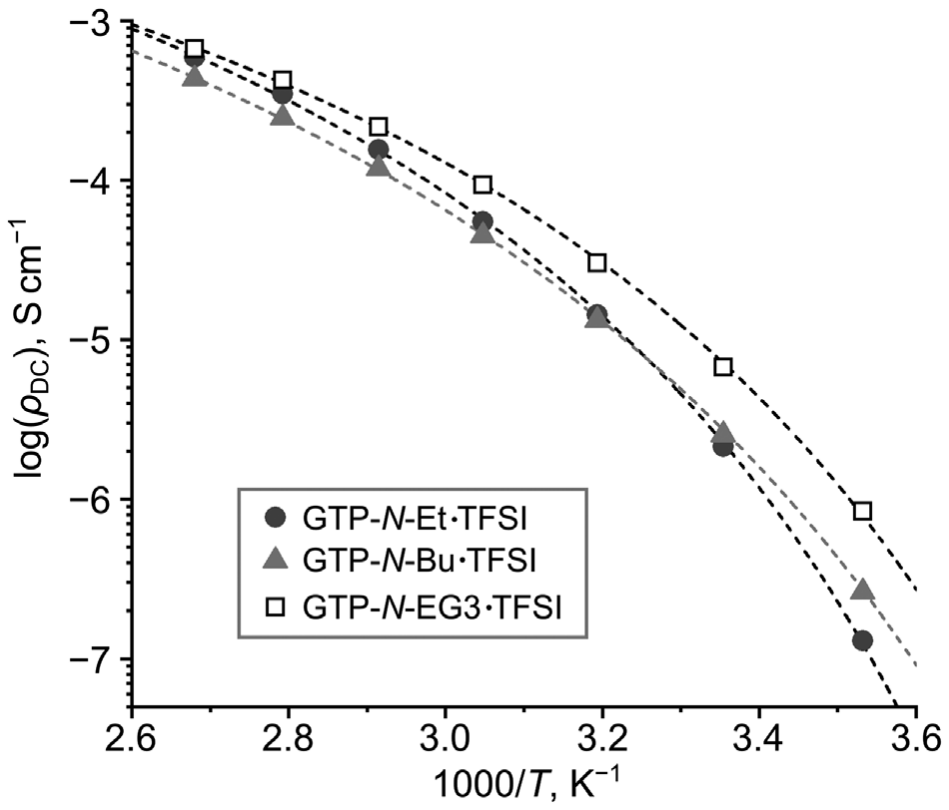
Temperature dependence of DC conductivity (*σ*_DC_). Dashed curves represent fits using Equation (1). Fitting parameters are provided in Table 3.

The ionic conductivity of the cationic GTPs was determined using impedance spectroscopy under dry conditions.^[^ ^46^ ^]^ The direct current conductivity (*σ*_DC_) was derived from the plateau region of the conductivity versus frequency plot (Figure S13, Supporting Information). The temperature dependence of the ionic conductivity for the cationic GTPs followed a Vogel–Fulcher–Tammann (VFT)‐type behavior (**Figure** **6**), indicating that ionic conduction is linked to the segmental motion of the polymer chains.^[^ ^22^, ^23^, ^24^, ^25^, ^26^, ^27^ ^]^ The data were analyzed using the equation

(1) $$\sigma_{\mathrm{DC}}=\sigma_{0}\times \exp\left\{-B/\left(T-T_{0}\right)\right\}$$

where *σ*_0_, *B*, and *T*_0_ are constants.^[^ ^22^ ^]^ The parameters obtained from the fit are summarized in **Table** **3**.

**Table 3:** Ionic conductivity of cationic GTPs and fitting parameters for Equation (1).

GTP	*σ*_DC_ (25 °C) [S cm^−1^]	*σ*_0_ [S cm^−1^]	*B*	*T*_0_ [K]
GTP‐*N*‐Et·TFSI	2.1 × 10^−6^	0.1233	778.2	226.8
GTP‐*N*‐Bu·TFSI	2.6 × 10^−6^	0.1467	933.8	212.4
GTP‐*N*‐EG3·TFSI	6.8 × 10^−6^	0.0791	729.0	219.8

The *σ*_DC_ values at 25 °C for the cationic GTPs were approximately 10^−6^ S cm^−1^. GTP‐*N*‐EG3·TFSI exhibited the highest ionic conductivity across all temperatures. This superior performance is likely due to its lowest *T*_g_ among the cationic GTPs. Using the fitting parameters and Equation (1), the *σ*_DC_ value for GTP‐*N*‐EG3·TFSI at 30 °C was calculated to be 1.2 × 10^−5^ S cm^−1^, higher than that of the reference polymer GTP‐(*N*‐Me)‐EG3·TFSI (7.5 × 10^−6^ S cm^−1^).^[^ ^36^ ^]^ Taking the fact that GTP‐*N*‐EG3·TFSI has higher *T*_g_ value (−24.2 °C) than GTP‐(*N*‐Me)‐EG3·TFSI (−37 °C) into account,^[^ ^36^ ^]^ this result looks to be strange, because the poly(ionic liquid)s with lower *T*_g_ usually exhibit higher ionic conductivity. Lower *T*_g_ value of GTP‐(*N*‐Me)‐EG3·TFSI than GTP‐*N*‐EG3·TFSI can be explained by the previously reported experimental results that the increasing the number of the side groups often decreases *T*_g_ value due to the shielding of interactions between the polymer main chains.^[^ ^22^, ^43^ ^]^ As discussed above with the NMR results, the sterically crowded environment around the triazolium unit in GTP‐(*N*‐Me)‐EG3·TFSI lowers the dynamics of the cationic unit. Presumably, the dynamics of the cationic unit might be more important to promote the ion conduction than the segmental dynamics of the other parts which determine the *T*_g_ value.

Conversely, the *σ*_DC_ value for GTP‐*N*‐Bu·TFSI at 30 °C (5.0 × 10⁻⁶ S cm^−1^) was comparable to that of the reference polymer GP‐Im‐Bu·TFSI (5.3 × 10^−6^ S cm^−1^ at 30 °C),^[^ ^37^ ^]^ reflecting similar steric structures which may influence ionic conductivity. The *T*_g_ values of GTP‐*N*‐Bu·TFSI and GP‐Im‐Bu·TFSI were also comparable (−14.6 and −12 °C, respectively).^[^ ^37^ ^]^ Compared to cationic GTPs with different alkyl substituents (*N*‐ethyl and *N*‐butyl), GTP‐*N*‐Et·TFSI showed a stronger temperature dependency than GTP‐*N*‐Bu·TFSI. At higher temperatures, the ionic conductivity of GTP‐*N*‐Et·TFSI exceeds that of GTP‐*N*‐Bu·TFSI, despite its higher *T*_g_.

For further analysis, the *σ*_DC_ value was decomposed into two components: the conducting ion concentration (*p*) and the conducting ion mobility (*µ*), using the electrode polarization model (Supporting Information).^[^ ^47^, ^48^, ^49^ ^]^ The *σ*_DC_ value is expressed as the product of the elemental charge (1.60 × 10^−19^ C), *p*, and *µ* values [Equation (2)].

(2) $$\sigma_{\mathrm{DC}}=e\cdot p\cdot \mu$$

**Figure 7:**
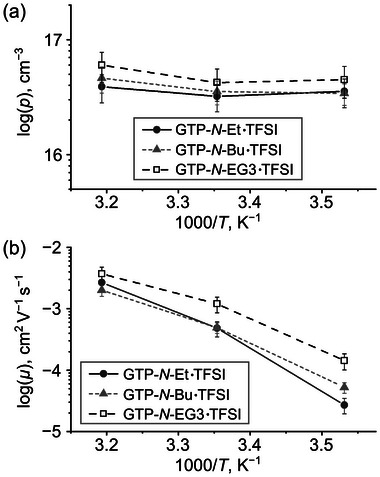
Temperature dependency of a) conducting ion concentration (*p*) and b) conducting ion mobility (*µ*) values.

**Figure** **7** illustrates the temperature dependence of the conducting ion concentration (*p*) and mobility (*µ*) values. The *p* values for all cationic GTPs were in the order of 10^16^ cm^−3^, which are relatively smaller compared to the previously reported cationic GTPs with *p*‐values on the order of 10^17^ cm^−3^.^[^ ^22^, ^25^, ^26^ ^]^ This difference is attributed to the positioning of the cationic unit; in this study, the cationic unit is located near the main chain, whereas in previous studies, it was at the end of side chains. Colby et al. reported that poly(ionic liquid)s with a long spacer between the main chain and the cationic unit exhibited higher conducting ion concentrations than those with an intermediate‐length spacer.^[^ ^50^ ^]^ The *N*‐EG3 substituent demonstrated a higher *p*‐value compared to the *N*‐alkyl substituents (Figure 7a), likely because the ethylene glycol chains enhance ion‐pair dissociation.^[^ ^20^, ^21^, ^51^ ^]^ In addition, the long, flexible ethylene glycol side chains soften the polymer matrix and facilitate ion diffusion, leading to a higher *µ* value than those of the *N*‐alkyl substituents (Figure 7b). From these *p‐* and *µ*‐values, it is evident that the different temperature dependencies of the *σ*_DC_ value between the *N*‐ethyl and *N*‐butyl substituents arise from the *µ* value's temperature dependency. Similar trends have been observed in other polymers, such as the dynamics of poly(alkyl methacrylate)s,^[^ ^52^ ^]^ diffusion of CH_4_ and CO_2_ molecules in the poly(alkyl acrylate) matrix,^[^ ^53^ ^]^ and the ionic conductivity of poly(ionic liquid)s.^[^ ^54^ ^]^ The shorter side chains provide less shielding of interactions between the polymer main chains, leading to a more significant decrease in polymer chain dynamics with temperature compared to longer side chains.

## 3 Conclusion

Cationic GTPs have been successfully synthesized through a thermal azide‐alkyne synthesis followed by decarboxylation and quaternization of the triazole unit. This research revisited the achievements of H. L. Cohen in preparing nonfunctionalized GTP (GTP‐H), highlighting that microwave‐assisted reactions are particularly effective for the decarboxylation of carboxyl‐functionalized GTP. The ionic conductivity of GTP‐*N*‐Bu·TFSI was found to be comparable to that of the reference polymer GP‐Im‐Bu·TFSI, while GTP‐*N*‐EG3·TFSI exhibited higher ionic conductivity than the reference polymer GTP‐(*N*‐Me)‐EG3·TFSI. These results suggested that reducing steric crowding around the triazolium unit is crucial for enhancing ionic conductivity. These findings contribute to the development of a facile synthesis for GTP‐based poly(ionic liquid)s with improved ionic conductive properties.

## 4 Experimental Section

### Synthesis of GTP‐COOH

PECH was cut into small pieces (<10 mm^3^) to fascinate the dissolution. PECH (3.0 g, 32 mmol monomer unit) and NaN_3_ (3.0 g, 46 mmol) were suspended in dry DMF (60 mL) in a 500‐mL round bottom flask. After 10 min N_2_ bubbling of the solution at room temperature with stirring, the mixture was stirred at 90 °C under N_2_ atmosphere for 24 h. After cooling to room temperature, the solution was diluted and centrifuged (5000 rpm, 10 min). The supernatant was vacuum‐filtrated through alumina powder (Al_2_O_3_ for column chromatography; Note: Alumina is inert to NaN_3_ under ordinary condition.).^[^ ^55^ ^]^ The solution was concentrated to the original volume (60 mL) with an evaporator. After 10 min N_2_ bubbling of the solution at room temperature with stirring, distilled propiolic acid (4.2 mL, 68 mmol) was added. The mixture was stirred at 50 °C under N_2_ atmosphere for 3 d. After cooling to room temperature, the solution was concentrated to the half volume with an evaporator. The solution was added dropwise in MeOH (600 mL) with stirring for precipitating the product. After rinsing with MeOH, the product was dried under vacuum overnight at 80 °C. The product was obtained as a white solid. Yield: 5.0 g (91%). ^1^H NMR (400 MHz, DMSO‐*d*_6_): *δ* = 3.00–4.10 (multiple broad peaks, 3H), 4.10–4.90 (multiple broad peaks, 2H), 8.01 (br, 0.12 H, triazole of 5‐functionalized product), 8.58 (br, 0.88 H, triazole of 4‐functionalized product); ^13^C NMR (100 MHz, DMSO‐*d*_6_): *δ* = 50.6, 67.0–69.0, 77.0, 129.6, 139.8, 161.8.

### Synthesis of GTP‐H

GTP‐COOH was grinded into powder with a mortar. In order to prevent over‐heating of the reaction solution by microwave irradiation, GTP‐COOH (1.0 g, 5.9 mmol) was completely dissolved in dry DMF (15 mL) at 60 °C with an aluminum heating/reaction block. After setting the solution to the microwave reactor, the reaction temperature was gradually raised with increasing the irradiation power (20 → 40 W) with stirring. The solution was allowed to react at 150 °C for 1 h. After cooling to room temperature, the solution was concentrated to 5 mL by evaporation. The solution was added dropwise to diethyl ether (100 mL) for precipitating the product with stirring in a 500‐mL PTFE beaker. After rinsing with diethyl ether, the product was dried under vacuum overnight at 80 °C. The product was obtained as a pale yellow solid. Yield: 0.65 g (88%). ^1^H NMR (400 MHz, DMSO‐*d*_6_): *δ* = 3.15–3.50 (br, overlapping to water peak), 3.73 (br, 1H), 4.20–4.48 (br, 2H), 7.70 (br, 1H), 8.02 (br, 1H); ^13^C NMR (100 MHz, DMSO‐*d*_6_): *δ* = 50.3, 68.1–68.9, 77.5, 125.9, 133.4.

### Synthesis of Cationic GTPs

As a representative of cationic GTPs, the synthetic procedure for GTP‐*N*‐Et·TFSI is described as follows. GTP‐H (0.61 g, 4.9 mmol) and iodoethane (2.0 mL, 25 mmol) were dissolved in dry DMF (6 mL). The mixture was stirred at 70 °C under N_2_ atmosphere for 24 h. After cooling to room temperature, distilled water was added (100 mL). The aqueous solution was washed with CH_2_Cl_2_ (100 mL × 3) by shaking in a separation funnel. For quick phase separation, the mixed solution was subjected to centrifugation (5000 rpm, 5 min). The organic layer was discarded. The aqueous layer was treated with activated carbon (5 g). After filtration, Li·TFSI (2.5 g/ 10 mL distilled water) was added to the solution with stirring. After 30 min, the product was recovered by centrifugation (5000 rpm, 5 min). The recovered product was dissolved in acetone. The acetone solution was concentrated with an evaporator (5 mL). The acetone solution was added dropwise to the aqueous solution containing Li·TFSI (2.5 g). After 30 min, the product was recovered by centrifugation (5000 rpm, 5 min). After rinsing with distilled water, the recovered product was dissolved in acetone. The solution was dried with MgSO_4_, filtrated and concentrated. The product was dried under vacuum overnight at 80 °C.

### GTP‐*N*‐Et·TFSI

^1^H NMR (400 MHz, CD_3_CN): *δ* = 1.61 (t, *J* = 7.4 Hz, 3H), 3.37–4.00 (br, 3H), 4.50–4.84 (m, 4H), 8.33 (m, 1H), 8.41 (m, 1H); ^13^C NMR (100 MHz, CD_3_CN): *δ* = 14.6, 50.7, 55.2, 67.9, 76.0–77.2, 120.8 (q, *J* = 319 Hz), 131.2, 132.6.

### GTP‐*N*‐Bu·TFSI

^1^H NMR (400 MHz, CD_3_CN): *δ* = 0.96 (t, *J* = 7.0 Hz, 3H), 1.39 (m, 2H), 1.96 (overlapping to acetonitrile), 3.37–4.00 (broad multiple peaks, 3H), 4.50–4.84 (overlapping multiple peaks, 4H), 8.34 (m, 1H), 8.40 (m, 1H); ^13^C NMR (100 MHz, CD_3_CN): *δ* = 13.6, 20.0, 31.8, 54.9, 55.3, 67.9, 76.4–77.4, 120.9 (q, *J* = 319 Hz), 131.5, 132.6.

### GTP‐*N*‐EG3·TFSI

^1^H NMR (400 MHz, CD_3_CN): *δ* = 3.28 (s, 3H), 3.35–3.85 (multiple peaks, 10H), 3.85–4.10 (overlapping peaks, 3H), 4.50–4.84 (overlapping multiple peaks, 4H), 8.36 (m, 1H), 8.59 (m, 1H); ^13^C NMR (100 MHz, CD_3_CN): *δ* = 54.9, 55.3, 58.9, 67.9, 68.4, 70.7, 70.8, 71.0, 72.5, 76.5–77.5, 120.9 (q, *J* = 319 Hz), 132.3, 132.6.

## Conflict of Interest

The authors declare no conflict of interest.

## Supporting information

Supporting Information

## Data Availability

The data that support the findings of this study are available in the Supporting Information of this article.
