# Supplementary material for: Copper‐Free Synthesis of Cationic Glycidyl Triazolyl Polymers
Source: Macromol Rapid Commun. 2024 Jul 2;45(24):2400416. doi: 10.1002/marc.202400416 (PMC11661660; doi:10.1002/marc.202400416)
Supplement: Supplementary file 1 — Supporting Information [file MARC-45-2400416-s001.pdf]

Macromolecular Rapid Communications

Supporting Information

for *Macromol. Rapid Commun.*, DOI 10.1002/marc.202400416

Copper-Free Synthesis of Cationic Glycidyl Triazolyl Polymers

*Taichi Ikeda**

Supporting Information

Copper-Free Synthesis of Cationic Glycidyl Triazolyl Polymers

*Taichi Ikeda**

Table of contents

1. Materials S2
2. Methods S2
3. ^{1}H and ^{13}C NMR spectra of GTPs S3
4. 2D NMR for peak assignment S8
5. Decarboxylation reaction of GTP-COOH S10
6. ^{1}H NMR spectra of GTP-(*N*-Me)-EG3·TFSI and GTP-*N*-EG3·TFSI S10
7. SEC chart of GTP-H S11
8. Impedance measurement S11
9. Electrode polarization model analysis S12
10. References S13

Correspondence Address
**Dr. Taichi Ikeda**<br><br>**E-mail: IKEDA.Taichi@nims.go.jp**<br><br>**Research Center for Macromolecules and Biomaterials**<br>**National Institute for Materials Science**<br>**Namiki 1-1, Tsukuba, 305-0044, JAPAN**

1. Materials

Polyepichlorohydrin (Average molecular weight: 700 kDa) was purchased from Scientific Polymer Products Inc. Propiolic acid, **iodoethane,** 1-iodobutane, triethylene glycol monomethyl ether, *p*-toluenesulfonyl chloride were purchased from Tokyo Chemical Industry. Sodium azide (NaN_3), sodium iodide (NaI), anhydrous magnesium sulfate ($MgSO_4$) and distilled water were purchased from Nacalai Tesque. Lithium bis(trifluoromethanesulfonyl)imide and dry solvents were purchased from Kanto Chemical. Alumina (activated, 200 mesh) was purchased from FUJIFILM Wako Chemicals. 2-[2-(2-Methoxyethoxy)ethoxy]ethyl iodide was prepared according to the literature.[S1]

2. Methods

Decarboxylation reaction of GTP-COOH was conducted with a microwave reactor (Discover SP, CEM Co.). Column chromatography was done by using Isolera Prime with SNAP Ultra flash chromatography cartridges (Biotage Co.). NMR spectra were recorded on a JEOL ECZ 400S (400 MHz and 100 MHz for ^1H and ^{13}C nuclei, respectively) with residual solvent as the internal standard. Size exclusion chromatography (SEC) was carried out at 50 °C with 0.01 M Li·NTf_2 in *N*, *N*-dimethylformamide (DMF) as the eluent on a Shimadzu Nexera XR with a Shim-pack GPC-80MD column. Polystyrene standards (PStQuick A and B, Tosoh Bioscience) were used for molecular weight calibration. Differential scanning calorimetry (DSC) was performed on Shimadzu DSC-60 Plus at a heating/cooling rate of 10 °C min^{-1} under N_2 flow. Thermogravimetric analysis (TGA) was performed with Shimadzu DTG-60 under N_2 flow. Ionic conductivity was measured using two-terminal impedance spectroscopy on a Solartron SI 1260 with a 1296 Dielectric Interface. The sample was placed on a disk-type blocking electrode (diameter: 5 mm) of a Solartron 12962A dielectric sample holder. The spacing between two electrodes was set to 300 μm. The frequency was swept from 1 MHz to 1 Hz by applying a sinusoidal voltage of 10 mV. The sample holder was placed inside a programmable thermostat chamber ESPEC SH-221 filled with dry N_2 gas. The sample was completely dried at 120 °C for 2 h before starting the measurement. The impedance data were collected from 100 to 10 °C at a cooling rate of 20 °C h^{-1} (0.33 °C min^{-1}). The data were processed utilizing ZView® Ver. 3.3c software.

3. ^{1}H and ^{13}C NMR spectra of GTPs

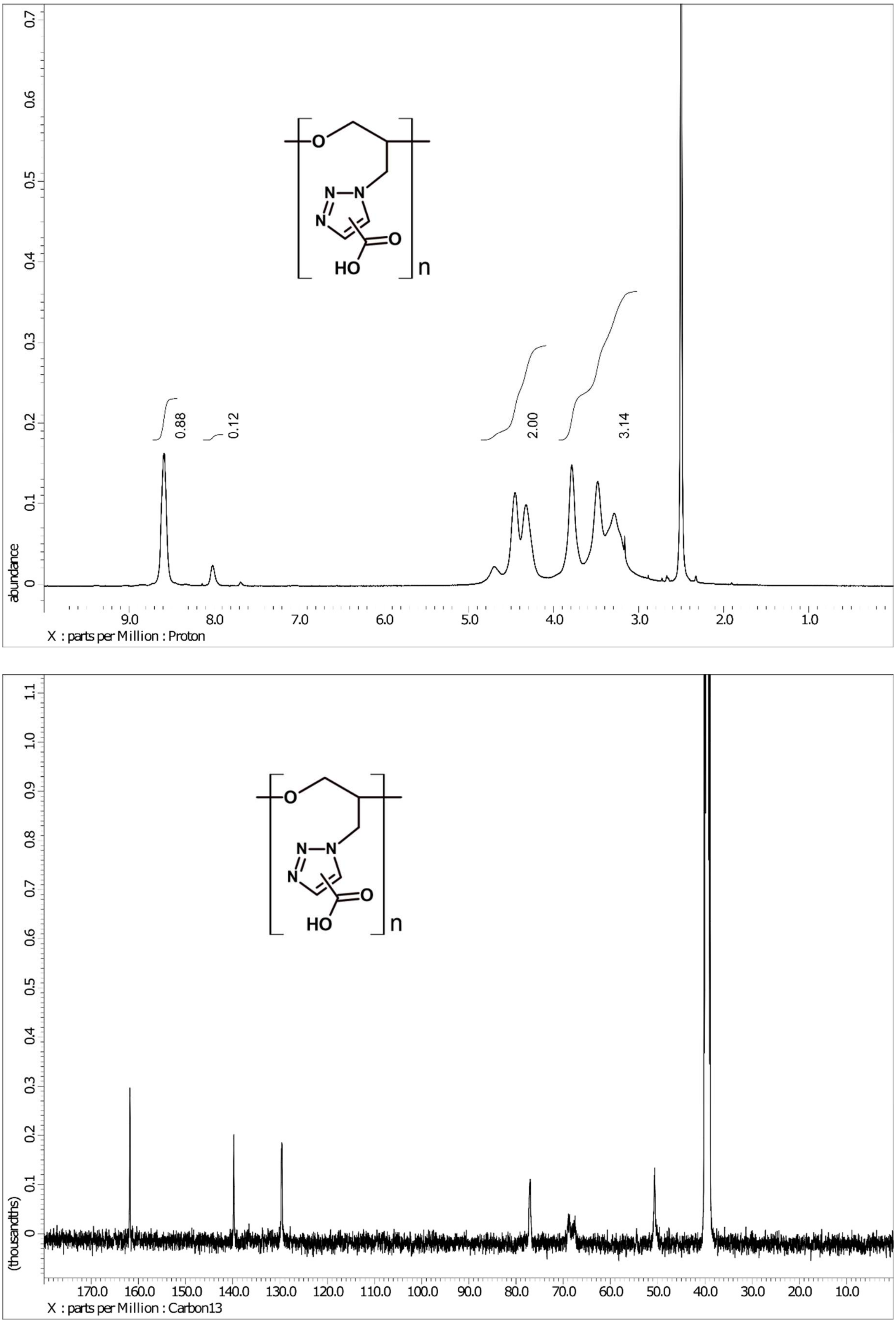

Figure S1. ^{1}H and ^{13}C NMR spectra of GTP-COOH (DMSO-d_6).

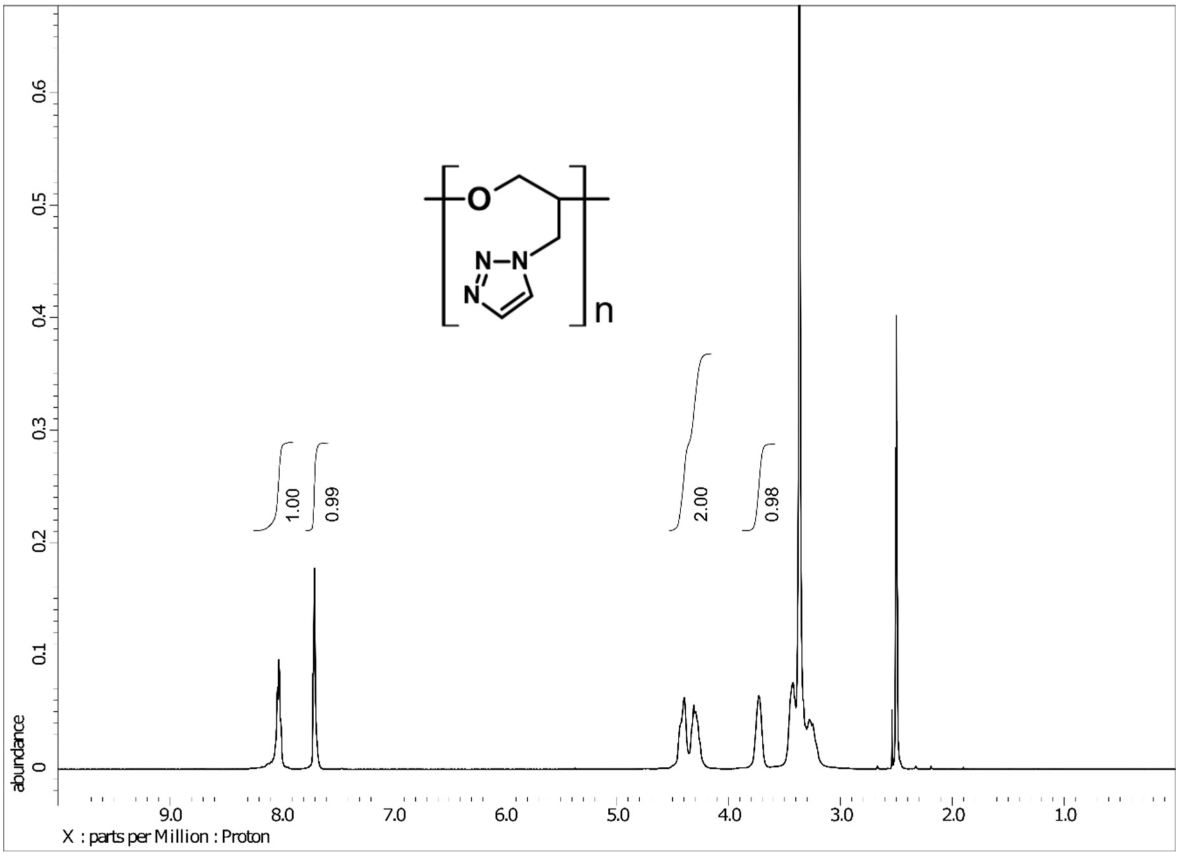

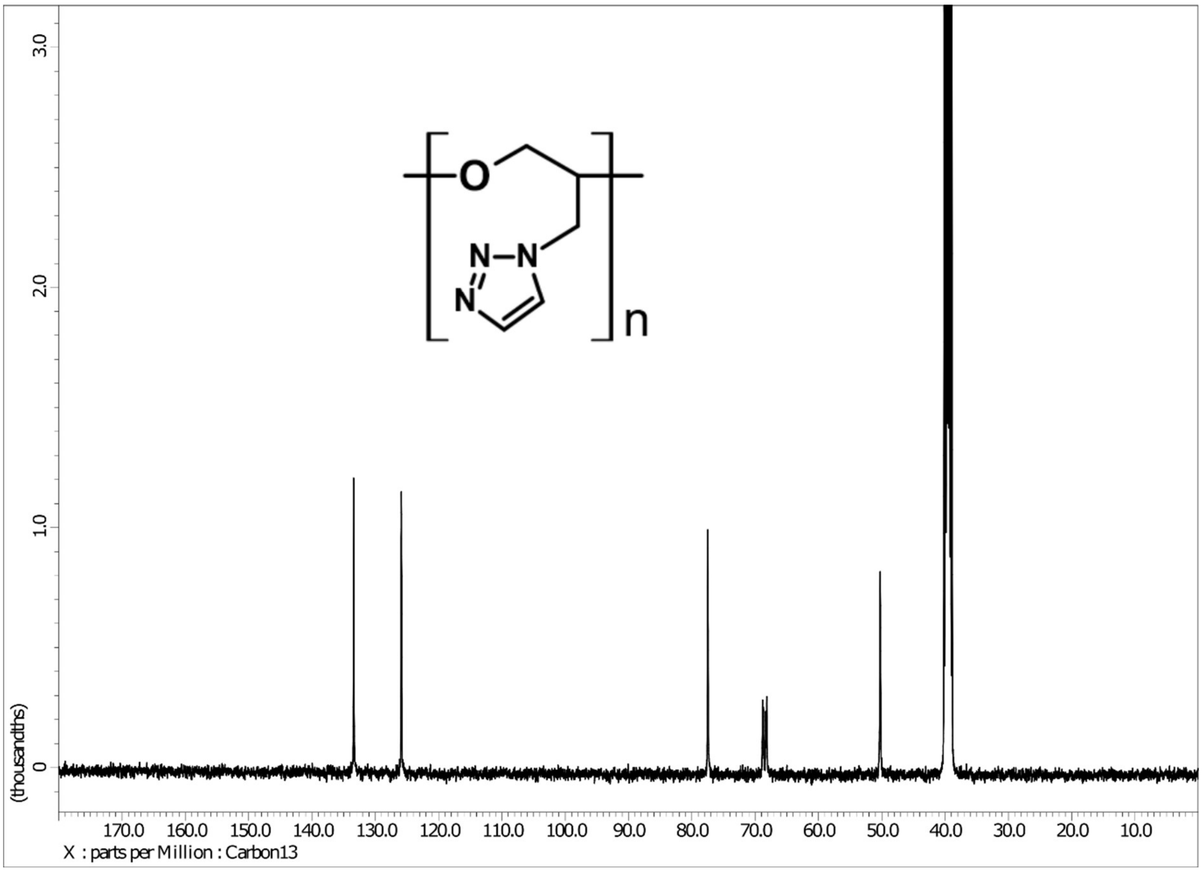

Figure S2. ^{1}H and ^{13}C NMR spectra of GTP-H (DMSO-d_6).

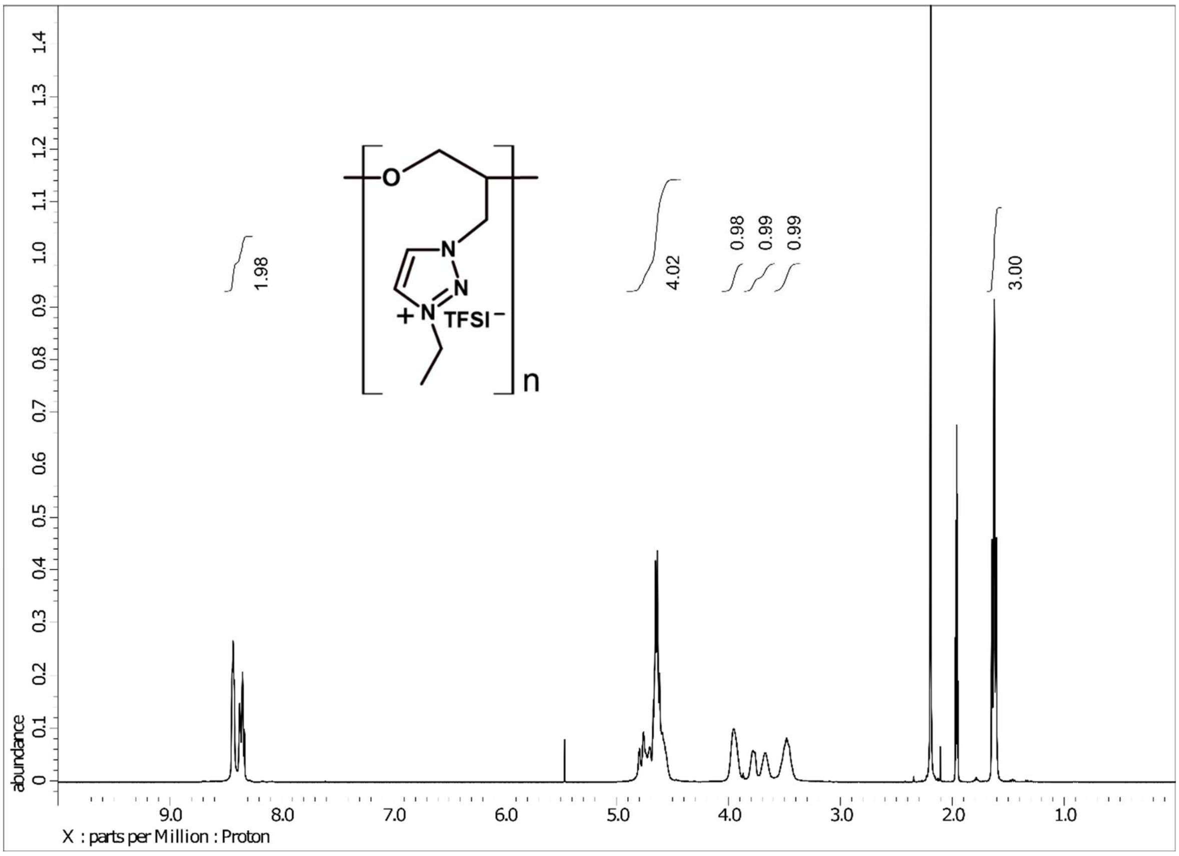

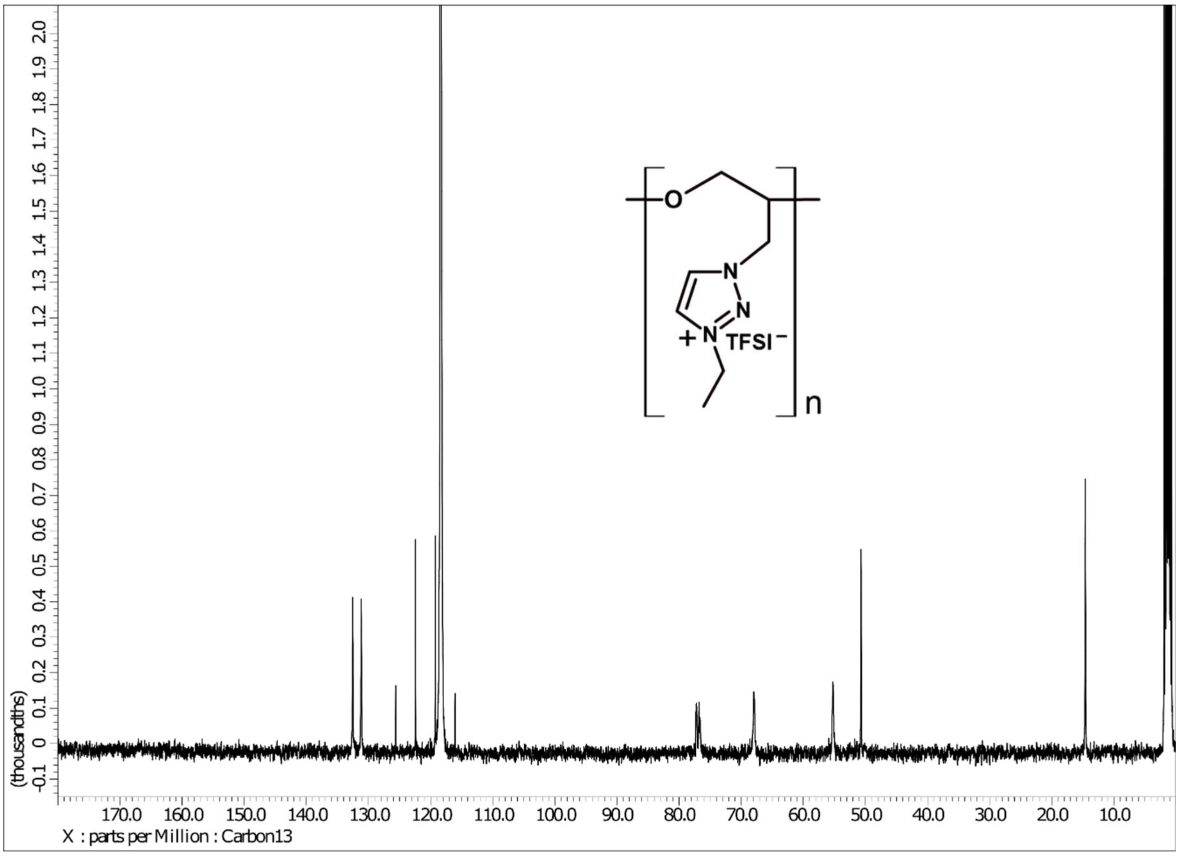

Figure S3. ^{1}H and ^{13}C NMR spectra of GTP-*N*-Et·TFSI (CD_3CN).

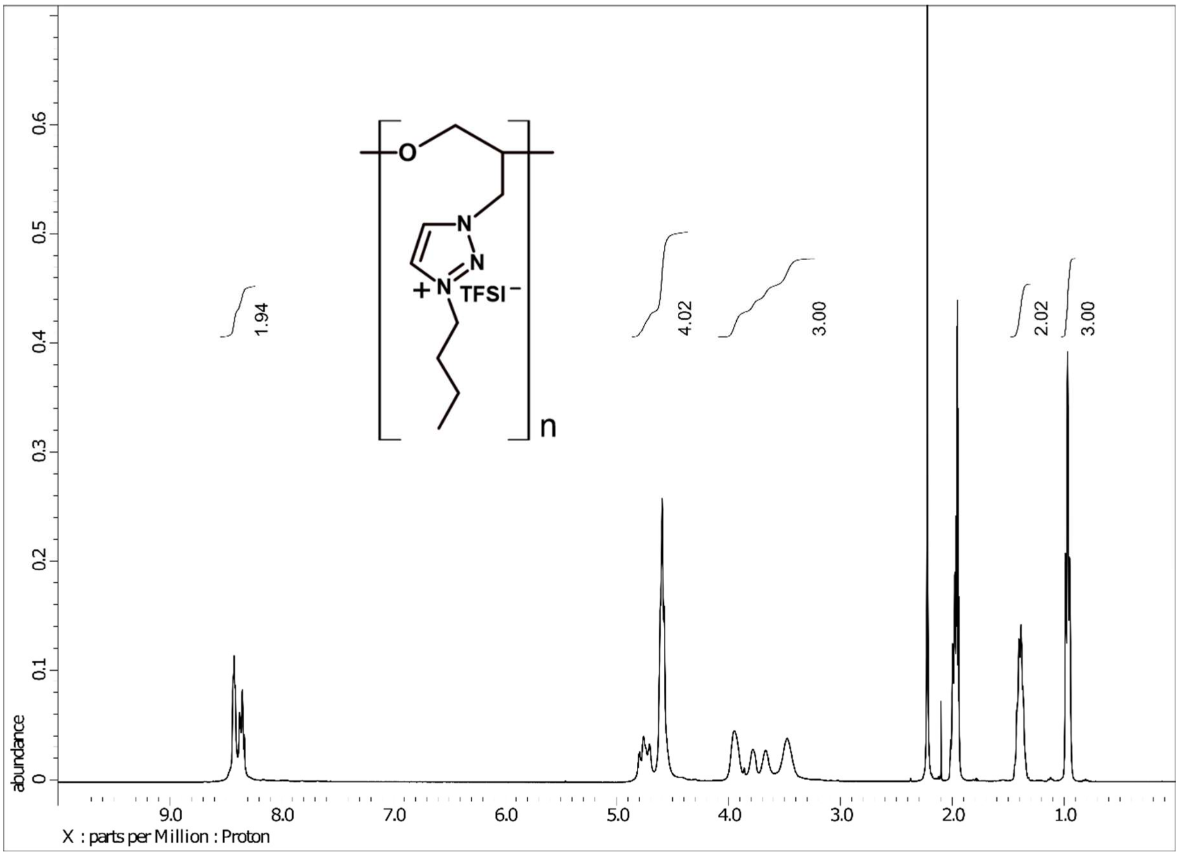

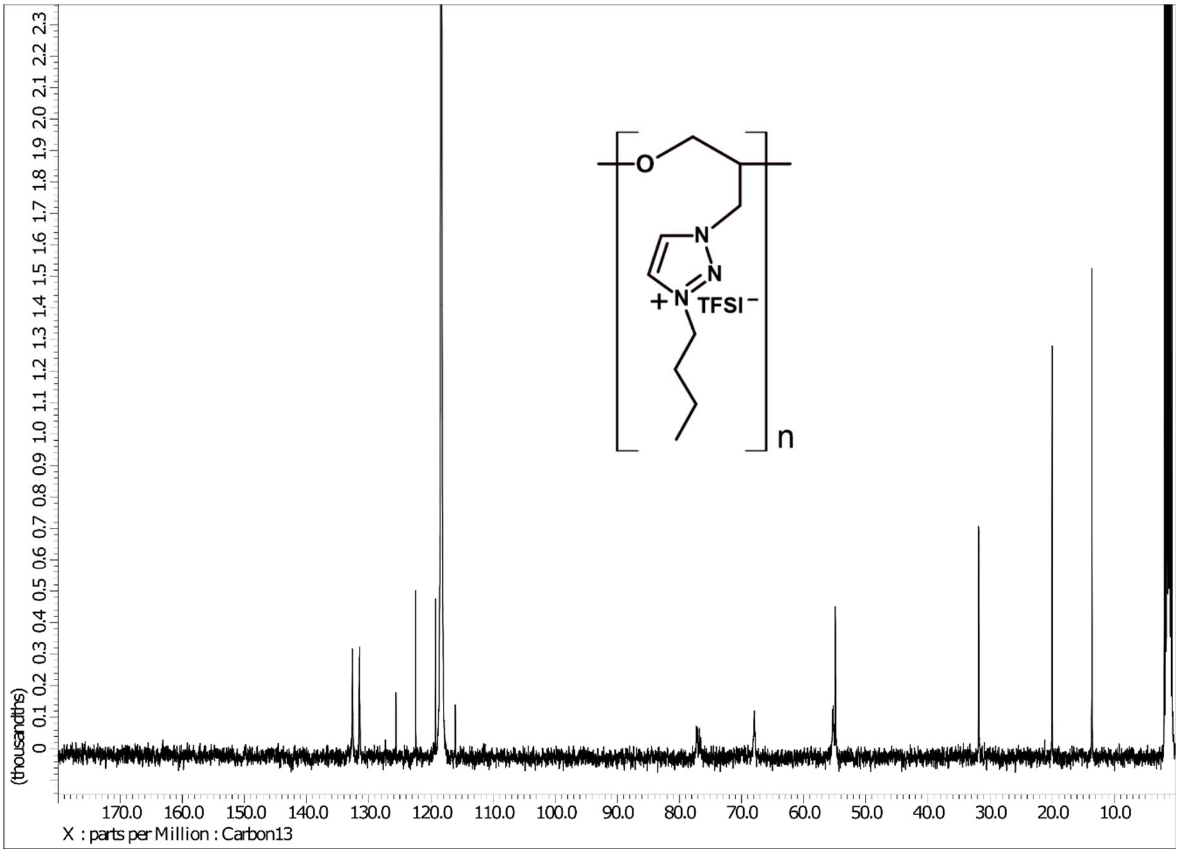

Figure S4. ^{1}H and ^{13}C NMR spectra of GTP-*N*-Bu·TFSI (CD_3CN).

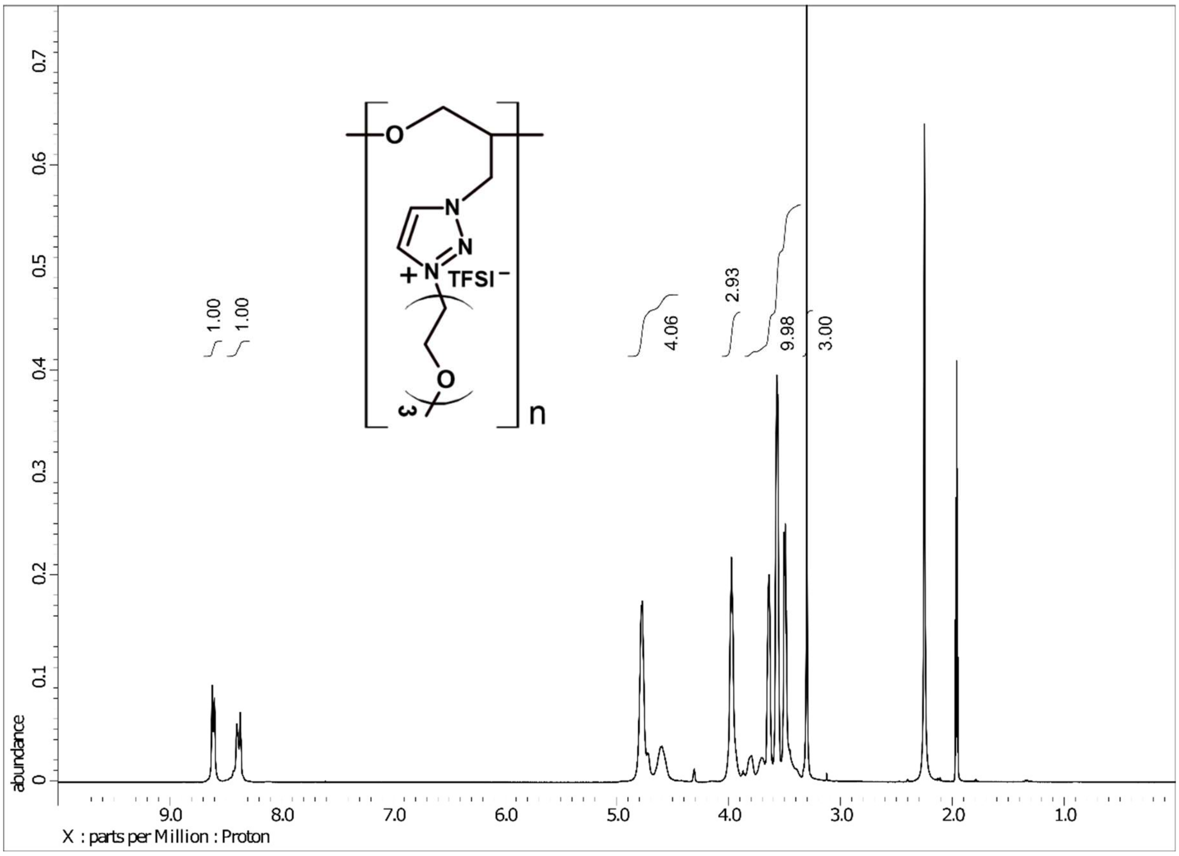

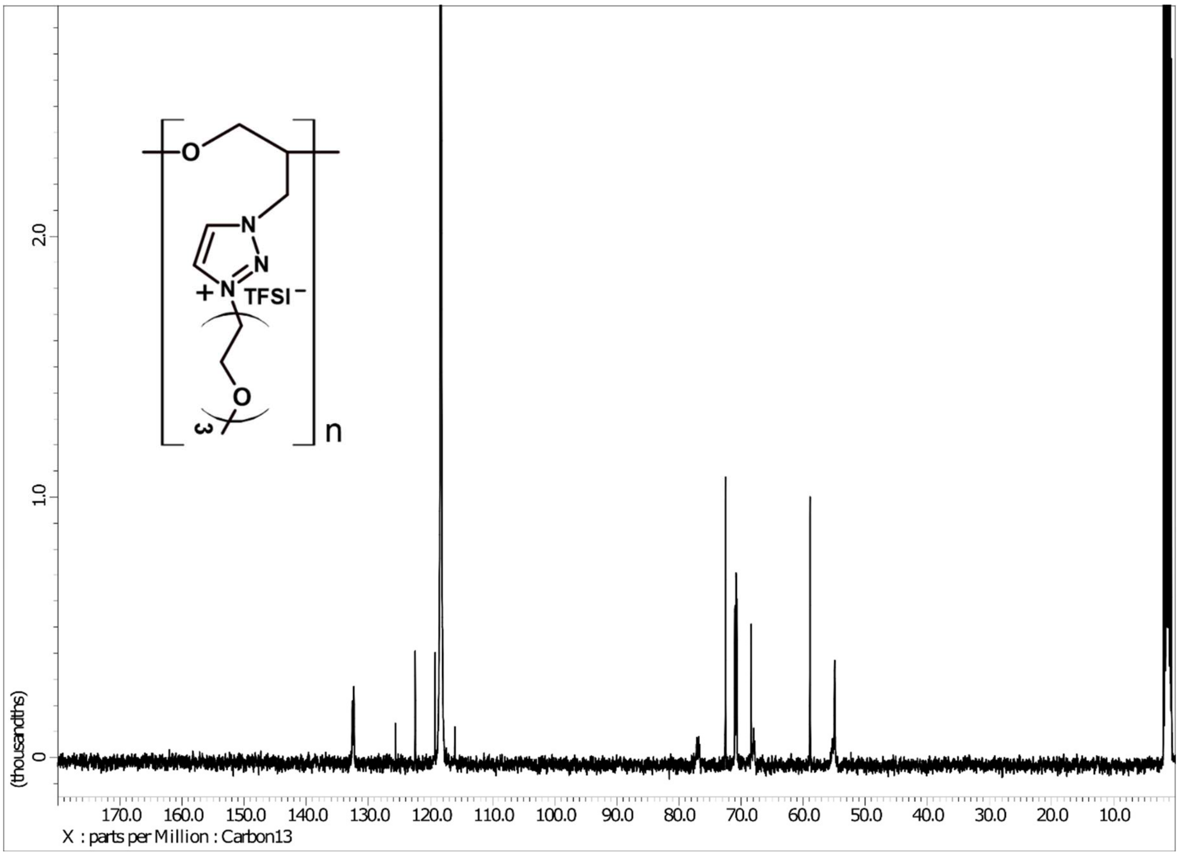

Figure S5. ^{1}H and ^{13}C NMR spectra of GTP-*N*-EG3·TFSI (CD_3CN).

4. 2D NMR for peak assignment

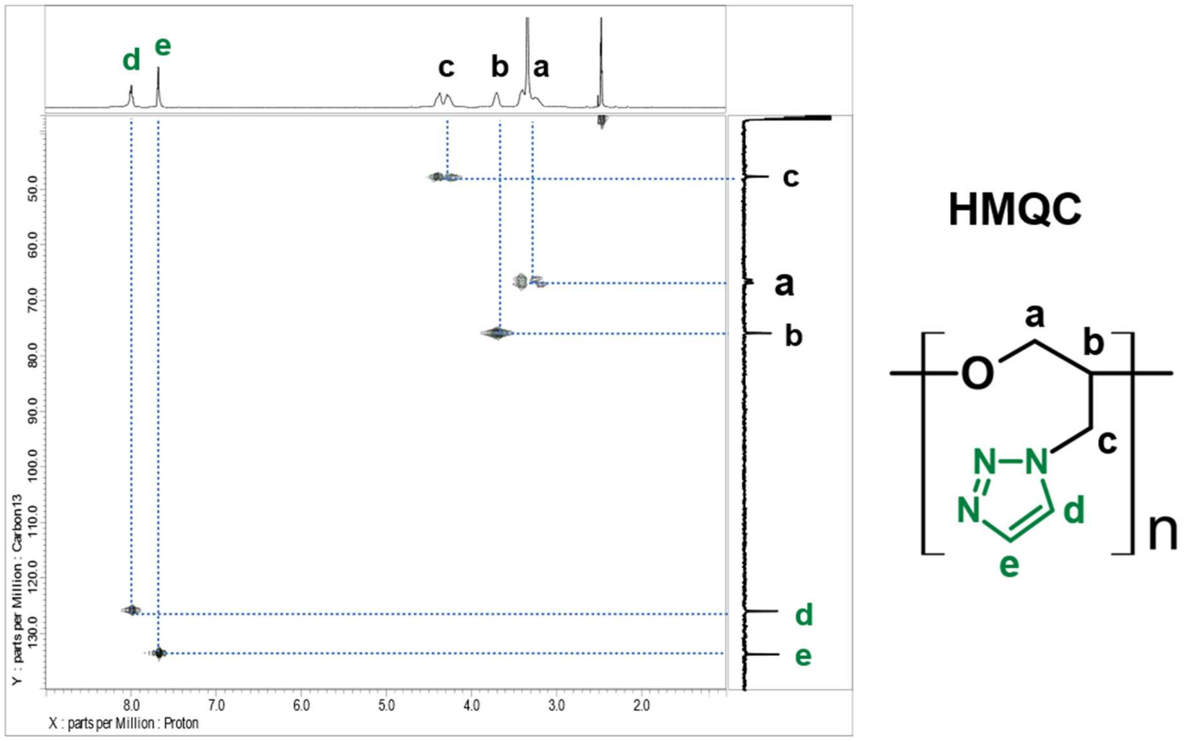

Figure S6. ^{1}H-^{13}C HMQC spectra of GTP-H (CD_3CN).

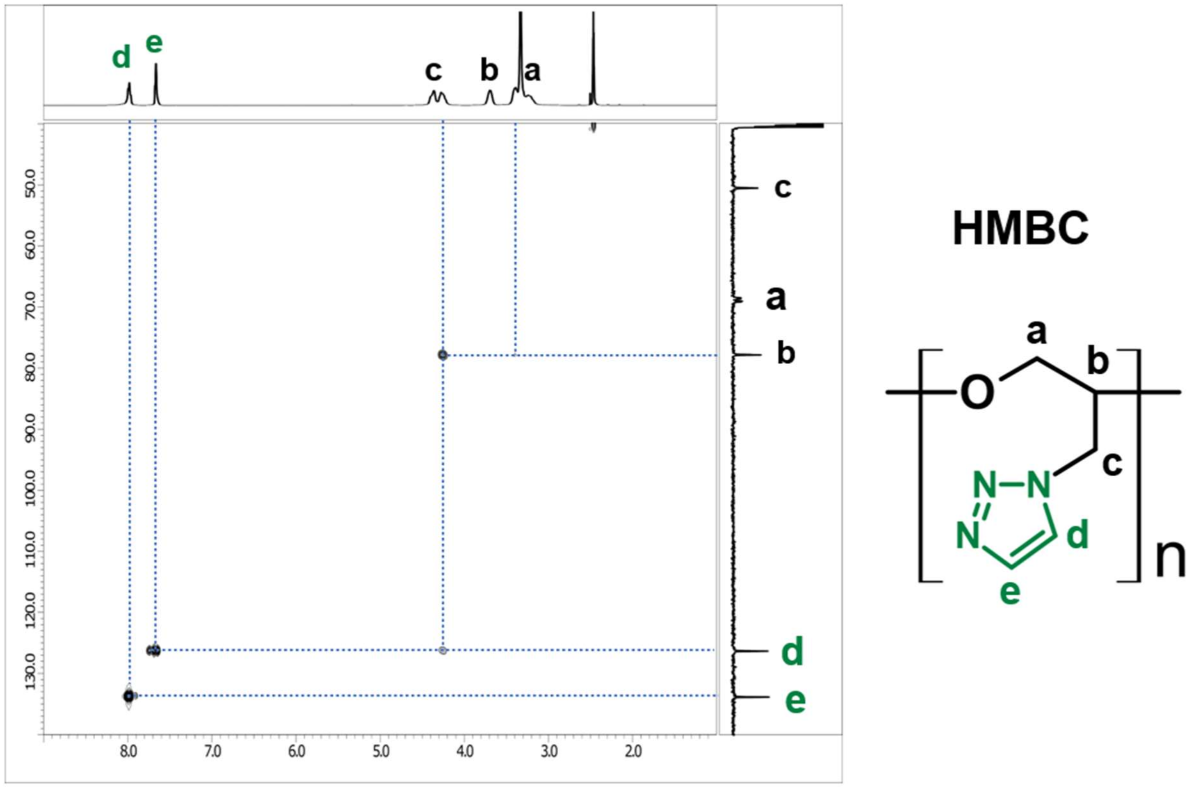

Figure S7. ^{1}H-^{13}C HMBC spectra of GTP-H (CD_3CN).

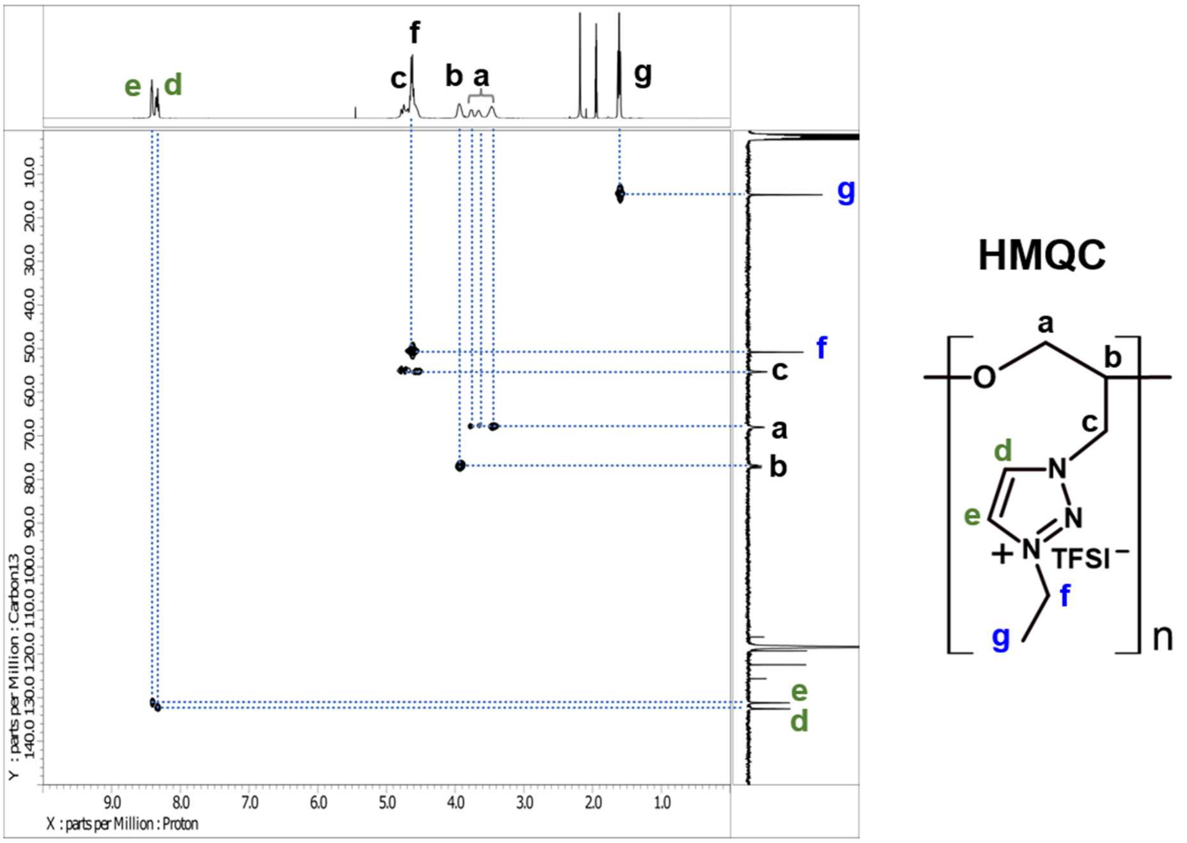

Figure S8. ^{1}H-^{13}C HMQC spectra of GTP-*N*-Et·TFSI (CD_3CN).

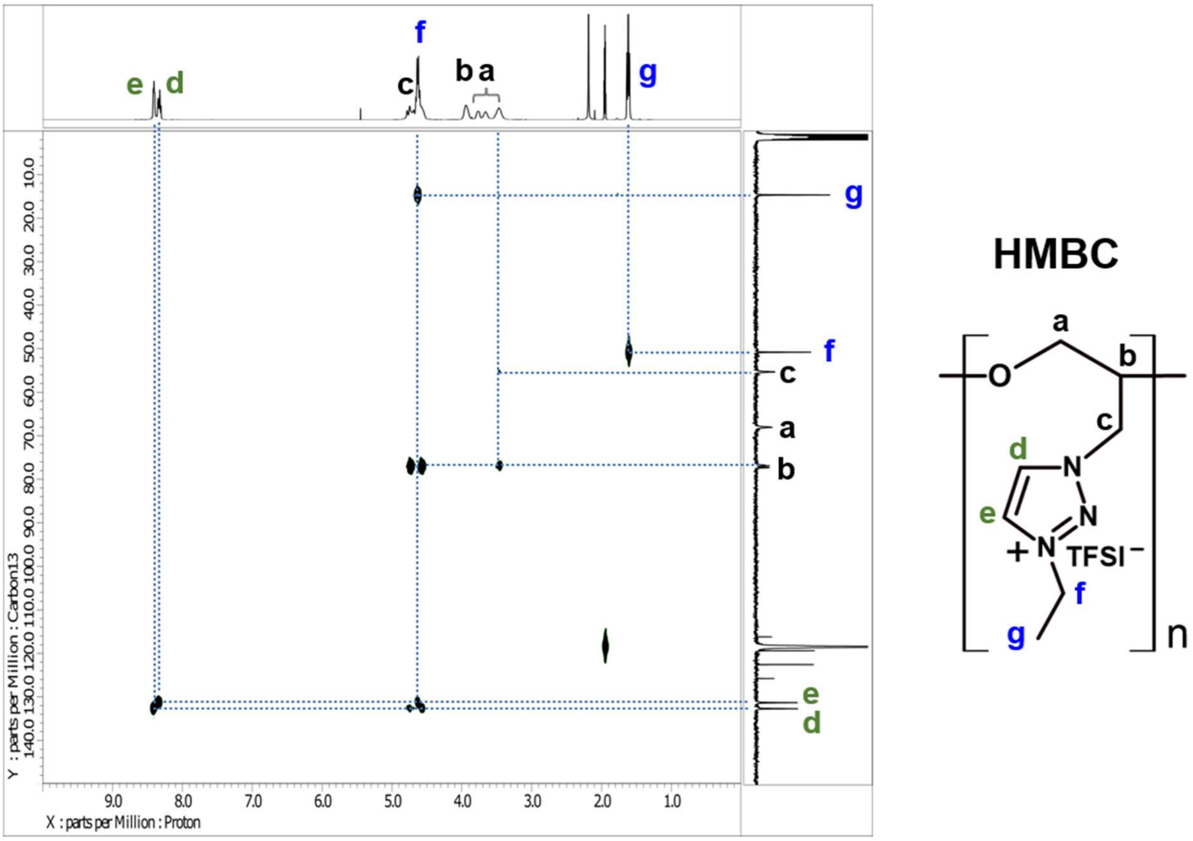

Figure S9. ^{1}H-^{13}C HMBC spectra of GTP-*N*-Et·TFSI (CD_3CN).

5. Decarboxylation reaction of GTP-COOH

GTP-COOH (0.50 g) was dissolved in dry DMF (5 mL). In case of microwave-assisted reaction, the solutions (0.8 mL) were reacted independently with different reaction time (5 min, 15 min, 30 min, 45 min and 60 min) at 150 ºC. On the other hand, the solution (5 mL) in a round-bottom flask was heated with an aluminum reaction block at 150 ºC. Then the samples (0.8 mL) were taken from the reaction solution at 1h, 2h, 4h and 8h. After the eveporation of the solvent, the samples were dissolved in DMSO-d_6 and characterized with ^{1}H NMR.

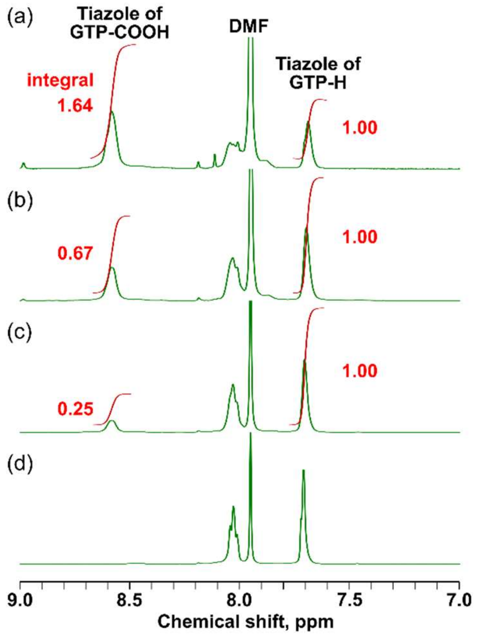

Figure S10. Changes in ^{1}H NMR spectrum during the decarboxylation of GTP-COOH conducted with an aluminum reaction heating block in DMF solution at 150 °C. Solvent: DMSO-d_6. Reaction times: (a) 1 h, (b) 2 h, (c) 4 h, (d) 8 h.

6. ^{1}H NMR spectra of GTP-(*N*-Me)-EG3·TFSI and GTP-*N*-EG3·TFSI

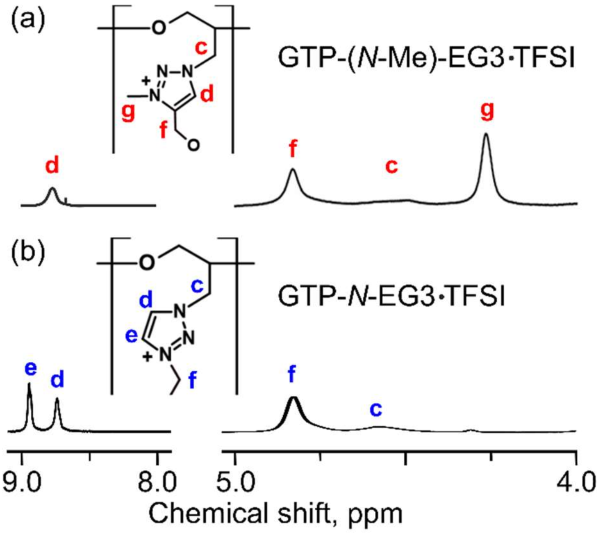

Figure S11. ^{1}H NMR spectra of (a) GTP-(*N*-Me)-EG3·TFSI (Reproduced from Ref. S2 with permission from the Royal Society of Chemistry) and (b) GTP-*N*-EG3·TFSI. Solvent: DMSO-d_6.

7. SEC chart of GTP-H

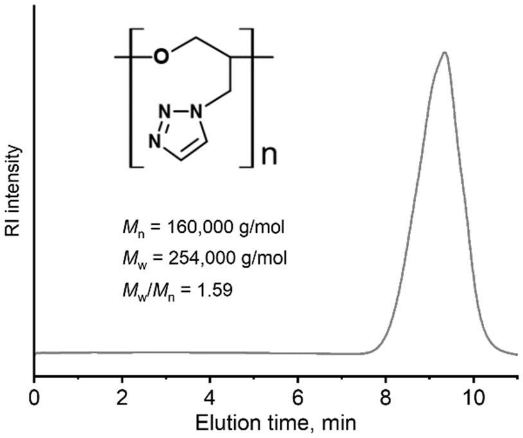

Figure S12. SEC chart of GTP-H. Solvent: 0.01 M Li·TFSI in DMF. Flow rate: 1.0 mL min^{-1}. Polystyrene standard.

8. Impedance measurement

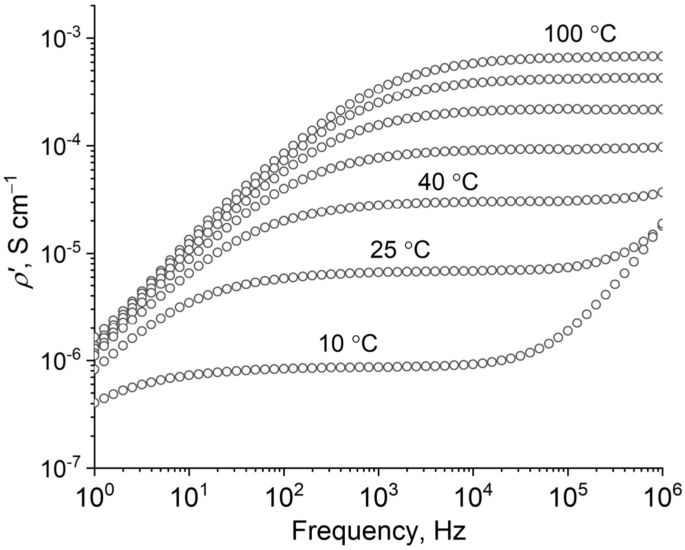

Figure S13. Conductivity vs frequency plot for GTP-*N*-EG3·TFSI. Dry condition.

Table S1. Ionic conductivity of cationic GTPs (Average ± standard error, n = 3)

Temp.	GTP-*N*-Et·TFSI		GTP-*N*-Bu·TFSI		GTP-*N*-EG3·TFSI	
°C	σ, S cm^{-1}	standard error	σ, S cm^{-1}	standard error	σ, S cm^{-1}	standard error
100	5.93×10^{-4}	1.9×10^{-5}	4.34×10^{-4}	4.9×10^{-6}	6.67×10^{-4}	6.4×10^{-6}
85	3.47×10^{-4}	1.1×10^{-5}	2.47×10^{-4}	1.9×10^{-6}	4.25×10^{-4}	1.3×10^{-6}
70	1.55×10^{-4}	4.2×10^{-6}	1.19×10^{-4}	9.4×10^{-7}	2.16×10^{-4}	7.4×10^{-7}
55	5.51×10^{-5}	1.4×10^{-6}	4.50×10^{-5}	3.9×10^{-7}	9.41×10^{-5}	4.0×10^{-7}
40	1.43×10^{-5}	3.8×10^{-7}	1.32×10^{-5}	1.5×10^{-7}	3.04×10^{-5}	1.2×10^{-7}
25	2.14×10^{-6}	6.4×10^{-8}	2.55×10^{-6}	6.0×10^{-8}	6.76×10^{-6}	6.8×10^{-8}
10	1.31×10^{-7}	6.3×10^{-9}	2.62×10^{-7}	1.1×10^{-8}	8.42×10^{-7}	1.9×10^{-8}

9. Electrode polarization model analysis

The Macdonald/Coelho model treats electrode polarization as a simple Debye relaxation with loss tangent (tan δ):[S3–S5]

$$\tan \delta = \omega\, \tau_{EP}/(1 + \omega^2 \tau_\sigma \tau_{EP}) \qquad \text{(S1)}$$

where τ_{EP} and τ_σ are the electrode polarization time and the conductivity time, respectively, and ω is the angular frequency ($2\pi f$). From the fitting of the data with equation (S1), we obtained the values of τ_{EP} and τ_σ (Fig. S16). The values of p and μ were then calculated from equations (S2) and (S3), respectively:

$$p = (1/(\pi l_B L^2))(\tau_{EP}/\tau_\sigma)^2 \qquad \text{(S2)}$$

$$\mu = eL^2\tau_\sigma/(4\tau_{EP}{}^2 kT) \qquad \text{(S3)}$$

where l_B is the Bjerrum length given by $e^2/(4\pi\tau_\sigma\sigma_{DC}kT)$, L is the spacing between electrodes, e is the elementary charge, k is the Boltzmann constant and T is the absolute temperature.

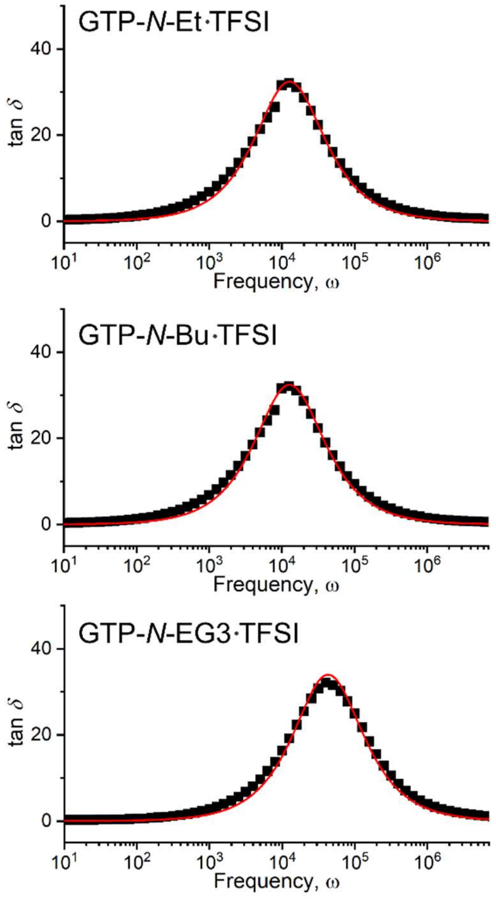

Figure S14. The plot of tan δ vs angular frequency at 25 °C under dry condition. The red curves were obtained from fitting by equation (S1).

Table S2. Conducting ion concentration of cationic GTPs (Average ± standard error, $n = 3$)

Temp.	GTP-*N*-Et·TFSI		GTP-*N*-Bu·TFSI		GTP-*N*-EG3·TFSI	
°C	cm^{-3}	standard error	cm^{-3}	standard error	cm^{-3}	standard error
40	3.90×10^{16}	1.1×10^{16}	4.62×10^{16}	1.2×10^{16}	6.02×10^{16}	1.8×10^{16}
25	3.22×10^{16}	8.6×10^{15}	3.53×10^{16}	8.3×10^{15}	4.24×10^{16}	1.3×10^{16}
10	3.55×10^{16}	9.9×10^{15}	3.41×10^{16}	7.3×10^{15}	4.48×10^{16}	1.4×10^{16}

Table S3. Conducting ion mobility of cationic GTPs (Average ± standard error, $n = 3$)

Temp.	GTP-*N*-Et·TFSI		GTP-*N*-Bu·TFSI		GTP-*N*-EG3·TFSI	
°C	$cm^2\ V^{-1}s^{-1}$	standard error	$cm^2\ V^{-1}s^{-1}$	standard error	$cm^2\ V^{-1}s^{-1}$	standard error
40	2.67×10^{-3}	7.3×10^{-4}	2.00×10^{-3}	4.1×10^{-4}	3.73×10^{-3}	1.0×10^{-3}
25	4.83×10^{-4}	1.3×10^{-4}	4.94×10^{-4}	9.5×10^{-5}	1.21×10^{-3}	3.4×10^{-4}
10	2.70×10^{-5}	7.5×10^{-6}	5.22×10^{-5}	1.0×10^{-5}	1.43×10^{-4}	4.2×10^{-5}

10. References

S1 H. R. Li, T. J. Jensen, F. R. Fronczek, M. G. H. Vicente, *J. Med. Chem.* **2008**, *51,* 502–511.

S2 M. M. Obadia, A. Jourdain, A. Serghei, T. Ikeda, E. Drockenmuller, *Polym. Chem.* **2017**, *8,* 910–917

S3 J. R. Macdonald, *Phys. Rev.* **1953**, *92,* 4–17.

S4 R. Coelho, *Rev. Phys. Appl.* **1983**, *18,* 137–146.

S5 R. J. Klein, S. H. Zhang, S. Dou, B. H. Jones, R. H. Colby, J. Runt, *J. Chem. Phys.* **2006**, *124*, 144903.
